# Supplementary material for: Functional SARS-CoV-2-sperific immune memory persists after mild COVID-19
Source: Res Sq. 2020 Aug 13:rs.3.rs-57112. Preprint. [Version 1] doi: 10.21203/rs.3.rs-57112/v1 (PMC7430600; doi:10.21203/rs.3.rs-57112/v1)
Supplement: Supplement [file PepperSI200810.pdf]

Supplemental Table 1

Marker	Flourochrome	Clone	Supplier
**Innate cell surface panel**			
CD16	BUV395	3G8	BD
Live/Dead Blue	BUV450	N/A	Thermo Fisher
CD15	BUV563	W6D3	BD
CD14	BUV615	M5E2	BD
CD56	BUV737	NCAM16.2	BD
CD31	BV421	WM59	Biolegend
HLADR	BV510	L243	Biolegend
CD11c	BV605	3.9	BioLegend
CD3	BV650	OKT3	Biolegend
CD19	BV650	HIB19	Biolegend
CD10	BV711	H110a	BioLegend
CD123	BV785	6H6	Biolegend
FCAR	FITC	A59	Biolegend
CD66b	PerCp Cy5.5	G10F5	Biolegend
CD9	PE	HI9a	Biolegend
CD163	PE-Cy7	GHI/61	Biolegend
CD41	APC	HIP8	BioLegend
CD304	APC-Fire	12C2	Biolegend
**B cell surface panel**			
Tetramer	PE		
Decoy	PE-Cy5		
CD19	FITC	HIB19	BD
CD20	P5.5	2H7	BD
CD3 (dump)	BV711	UCHT1	BD
CD14 (dump)	BV711	MϕP9	BD
CD16 (dump)	BV711	3G8	BD
IgM	BV510	MHM-88	BioLegend
IgD	PE-Cy7	IA6-2	BD
IgG	BV786	G18-145	BD
CD21	SB600	HB5	Thermo
CD27	BV421	M-T271	BioLegend
CD38	AF700	HIT2	BD
IgA	Biotin	IS11-8E10	Miltenyi
Streptavidin	BUV395		BD
**B cell intracellular panel**			
Tetramer	PE		
Decoy	PE-Cy5		
CD19	FITC	HIB19	BD
CD20	P5.5	2H7	BD
CD3 (dump)	APC-Cy7	HIT3a	BioLegend
CD14 (dump)	APC-Cy7	M5E2	BIoLegend
CD16 (dump)	APC-Cy7	3G8	BD
IgM	BV510	MHM-88	BioLegend
IgD	PE-Cy7	IA6-2	BD
IgG	BV786	G18-145	BD
CD21	SB600	HB5	Invitrogen
CD27	BV711	M-T271	BioLegend
CD38	AF700	HIT2	BD
Live/dead	ef780		ThermoFisher
FCLR5	EF660	509F6	Invitrogen
CD11c	PEDazzle594	3.9	BioLegend
Tbet	BV421	4B10	Biolegend
Ki67	BUV395	B56	BD
**T cell Phenotyping Panel**			
Ki-67	BUV395	B56	BD
Live/Dead Blue	BUV496	N/A	ThermoFisher
CD45RA	BUV737	HI100	BD
CXCR5	BV421	RF8B2	BD
CD3	BV510	UCHT1	BD
CD4	BV605	SK3	BioLegend
CCR6	BV650	11A9	BD
CD19	BV711	SJ25C1	BD
CD16	BV711	3G8	BD
CD14	BV711	M5E2	BD
CD8	BV750	SK1	BD
CCR7	BV785	G04387	BioLegend
FoxP3	AF488	259D/C7	BD
ICOS	Percp-eF710	ISA-3	ThermoFisher
Bcl-6	PE	7D1	BioLegend
Blimp-1	PE CF594	6D3	BD
CXCR3	PE Cy5	IC6/CXCR3	BD
T-bet	PE Cy7	4B10	BioLegend
PD-1	APC	EH12, 2H7	BioLegend
CD38	AF700	HIT2	BD
HLA-DR	APC H7	L243	BioLegend

Marker	Flourochrome	Clone	Supplier
**T cell surface panel**			
**Marker**	**Flourochrome**	**Clone**	**Supplier**
CD3	BUV395	SK7	BD
CD45RA	BUV737	HI100	BD
CXCR3	BV421	BVD2-21C11	BD
CCR7	BV605	G04347	BioLegend
CCR6	BV650	OKT4	BioLegend
CD4	BV711	OKT4	BioLegend
CD127	BV786	hIL7Rm21	BD
pan-gdTCR	PerCP/ef710	B1	BioLegend
CCR10	PE	REA173	R&D
CCR4	PE/Dazzle594	L291H4	BioLegend
CD25	PE/Cy5	BC96	BioLegend
CD8a	PE/Cy7	SK1	ThermoFisher
CD19	Ax700	SJ25C1	ThermoFisher
LiveDead	APC/ef780	n/a	ThermoFisher
**T cell cytokine panel**			
**Marker**	**Flourochrome**	**Clone**	**Supplier**
CD3	BUV395	SK7	BD
Live/Dead Blue	BUV496	n/a	ThermoFisher
CD45RA	BUV737	HI100	BD
CD8a	BV510	SK1	BioLegend
CD19	BV605	SJ25C1	BioLegend
CCR7	BV605	G04347	BioLegend
CD4	BV650	OKT4	BioLegend
IL-4	FITC	MP4-25D2	BioLegend
IL-13	PE	JES10-5A2	BioLegend
IL17a	PE/Dazzle594	BL168	BioLegend
IL-22	PE/Cy7	22URTI	ThermoFisher
IFNg	Ax700	4S.B3	BioLegend
pan-gdTCR	APC/Fire75	B1	BioLegend
**T cell sort and restimulation panel**			
**Marker**	**Flourochrome**	**Clone**	**Supplier**
CD45RA	BUV737	HI100	BD
CXCR3	BV421	BVD2-21C11	BD
CD8a	BV510	SK1	BioLegend
CCR7	BV605	G04347	BioLegend
CCR6	BV650	OKT4	BioLegend
CD4	BV711	OKT4	BioLegend
CXCR5	BB515	RF8B2	BD
pan-gdTCR	PerCP/ef710	B1	BioLegend
CCR10	PE	REA173	R&D
CCR4	PE/Dazzle594	L291H4	BioLegend
CD25	PE/Cy5	BC96	BioLegend
CD127	PE/Cy7	hIL7Rm21	ThermoFisher
CPD	e670	n/a	ThermoFisher
CD19	Ax700	SJ25C1	ThermoFisher
LiveDead	APC/ef780	n/a	ThermoFisher
**T cell ex vivo stim panel**			
CD69	BUV395	FN50	BD
Live/Dead Blue	BUV496	N/A	ThermoFisher
CD45RA	BUV737	HI100	BD
CXCR5	BV421	RF8B2	BD
CD3	BV510	UCHT1	BD
CD4	BV605	SK3	BioLegend
CCR6	BV650	11A9	BD
CD19	BV711	SJ25C1	BD
CD16	BV711	3G8	BD
CD14	BV711	M5E2	BD
CD8	BV750	SK1	BD
CCR7	BV785	G04387	BioLegend
CD40L	FITC	24-31	ThermoFisher
ICOS	Percp-eF710	ISA-3	ThermoFisher
IL-21	PE	eBio3A3-N2	ThermoFisher
IL-17A	PE Dz594	BL168	Biolegend
CXCR3	PE Cy5	IC6/CXCR3	BD
IL-4	PE Cy7	MP4-25D2	BioLegend
IL-6	AF647	MQ2-13A5	BioLegend
IL-2	AF700	MQ1-17H12	BioLegend
IFNy	APC H7	4S.B3	BioLegend
**Mouse B cell panel**			
**Marker**	**Flourochrome**	**Clone**	**Supplier**
RBD tetramer			
Decoy tetramer			
CD4	FITC	GK1.5	BD
CD8	FITC	53.67	BD
B220	BV711	RA3-6B2	BD
CD138	BV605	281-2	BD
CD38	AF700	90	ThermoFisher
GI7	ef450	GL-7	ThermoFisher

Supplemental Table 1. Antibody information for flow cytometry experiments
