# Supplementary figures and images for: Functional SARS-CoV-2-sperific immune memory persists after mild COVID-19
Source: Res Sq. 2020 Aug 13:rs.3.rs-57112. Preprint. [Version 1] doi: 10.21203/rs.3.rs-57112/v1 (PMC7430600; doi:10.21203/rs.3.rs-57112/v1)

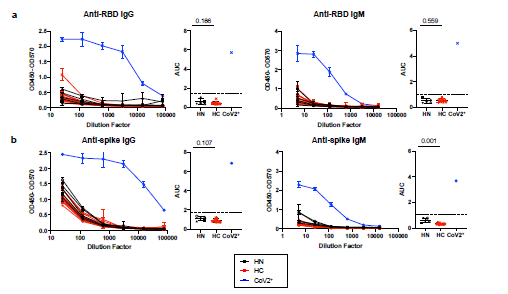

Supplement: Supplement [file FigS1.png]

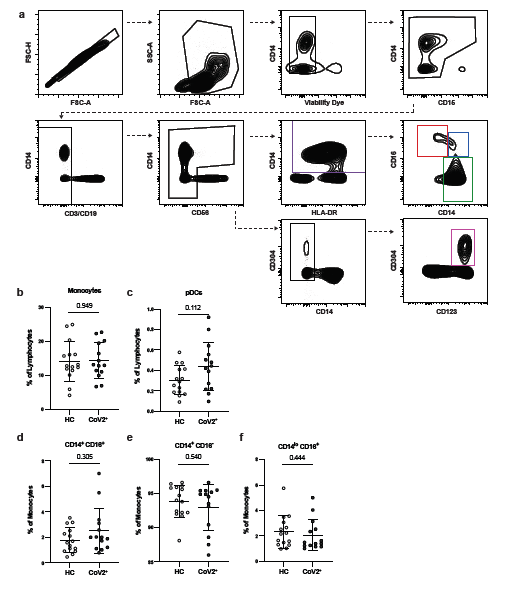

Supplement: Supplement [file FigS2.png]

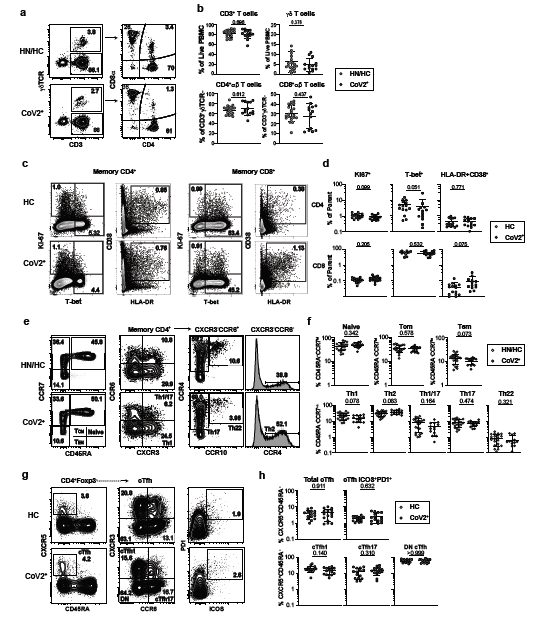

Supplement: Supplement [file FigS3.png]

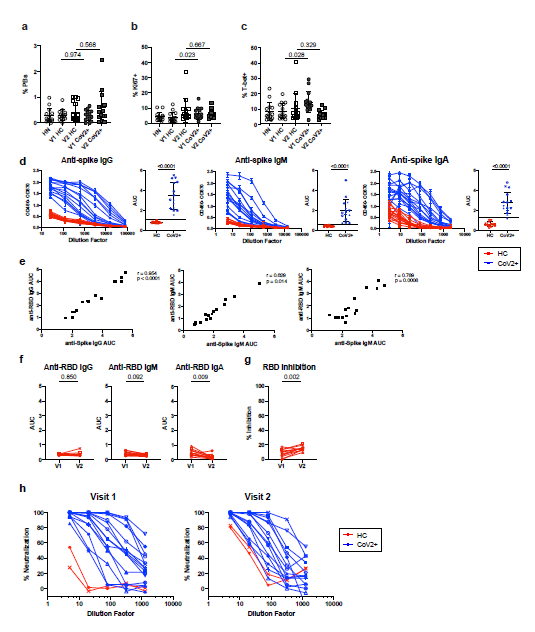

Supplement: Supplement [file FigS4.png]

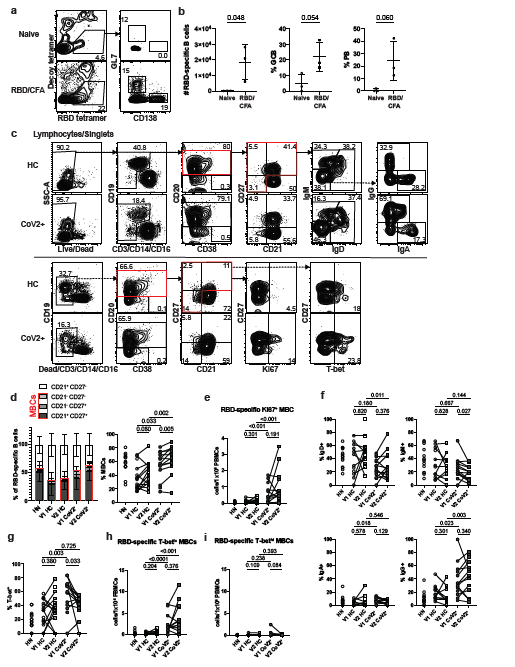

Supplement: Supplement [file FigS5.png]

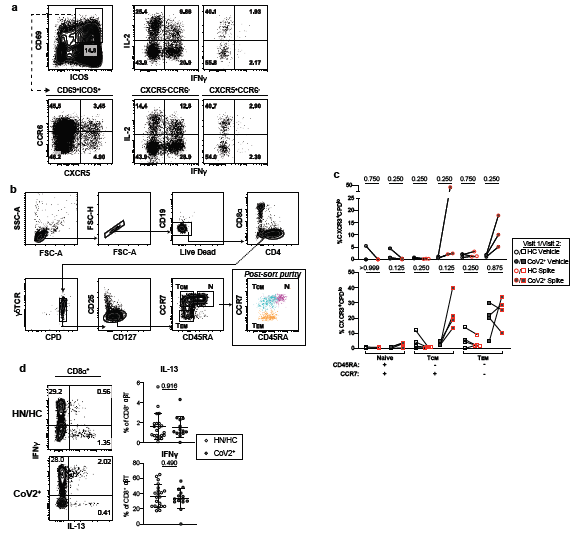

Supplement: Supplement [file FigS6.png]

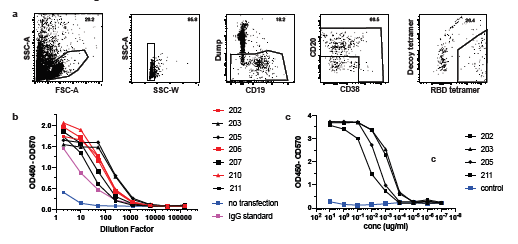

Supplement: Supplement [file FigS7.png]

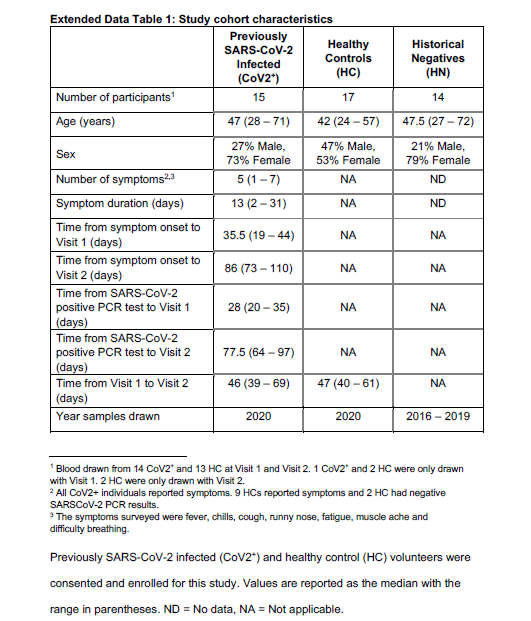

Supplement: Supplement [file TableS1.png]

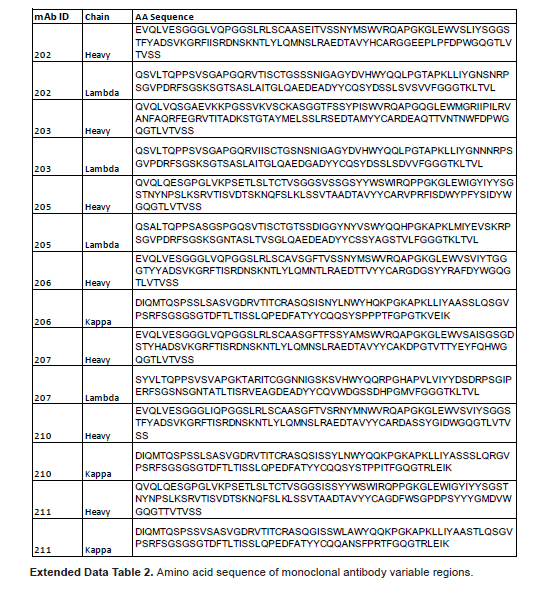

Supplement: Supplement [file TableS2.png]
